# Psychrophilic Yeasts: Insights into Their Adaptability to Extremely Cold Environments

**DOI:** 10.3390/genes14010158

**Published:** 2023-01-06

**Authors:** Haisheng Liu, Guiliang Zheng, Zhongwei Chen, Xiaoya Ding, Jinran Wu, Haili Zhang, Shulei Jia

**Affiliations:** 1 College of Agriculture and Bioengineering, Heze University, Heze 274000, China; 2 College of Marine Life Science, Ocean University of China, Qingdao 266100, China; 3 Nantong Ocean Centre of the Ministry of Natural Resources, Nantong 226002, China; 4 Institute of Microbiology, Chinese Academy of Sciences, Beijing 100101, China

**Keywords:** psychrophilic and mesophilic yeast, comparative genomics, fatty acid desaturase, antifreeze/ice-structuring proteins, chilling tolerance analysis

## Abstract

Psychrophilic yeasts are distributed widely on Earth and have developed adaptation strategies to overcome the effect of low temperatures. They can adapt to low temperatures better than bacteriophyta. However, to date, their whole-genome sequences have been limited to the analysis of single strains of psychrophilic yeasts, which cannot be used to reveal their possible psychrophilic mechanisms to adapt to low temperatures accurately and comprehensively. This study aimed to compare different sources of psychrophilic yeasts at the genomic level and investigate their cold-adaptability mechanisms in a comprehensive manner. Nine genomes of known psychrophilic yeasts and three representative genomes of mesophilic yeasts were collected and annotated. Comparative genomic analysis was performed to compare the differences in their signaling pathways, metabolic regulations, evolution, and psychrophilic genes. The results showed that fatty acid desaturase coding genes are universal and diverse in psychophilic yeasts, and different numbers of these genes exist (delta 6, delta 9, delta 12, and delta 15) in the genomes of various psychrophilic yeasts. Therefore, they can synthesize polyunsaturated fatty acids (PUFAs) in a variety of ways and may be able to enhance the fluidity of cell membranes at low temperatures by synthesizing C18:3 or C18:4 PUFAs, thereby ensuring their ability to adapt to low-temperature environments. However, mesophilic yeasts have lost most of these genes. In this study, psychrophilic yeasts could adapt to low temperatures primarily by synthesizing PUFAs and diverse antifreeze proteins. A comparison of more psychrophilic yeasts’ genomes will be useful for the study of their psychrophilic mechanisms, given the presence of additional potential psychrophilic-related genes in the genomes of psychrophilic yeasts. This study provides a reference for the study of the psychrophilic mechanisms of psychrophilic yeasts.

## 1. Introduction

Most of the Earth’s biosphere, both marine and terrestrial, is periodically or permanently exposed to temperatures lower than 5 °C. Although strongholds that are largely exposed to low temperatures are considered inhospitable to organisms, a tremendous amount of cold-adapted microorganisms have managed to survive in these low-temperature environments [1]. These cold-adapted microorganisms have been subdivided into psychrophilic microorganisms, which grow optimally at less than 15 °C, and psychrotolerant microorganisms, which survive at temperatures below 0 °C [2,3,4]. Previous studies have discovered the presence of culturable psychrophilic yeasts in low-temperature environments, and these yeasts can adapt to low temperatures better than bacteriophyta [5]. These psychrophilic yeasts have developed adaptation strategies to overcome the effect of low temperatures, freeze–thawing, intense radiation, oligotrophic factors, and extreme factors. Although lipids, enzymes, trehalose, heat kinin, and antifreeze proteins (AFPs) have been reported to be involved in the adaptation of psychrophilic yeasts to low temperatures, the related adaptation mechanism still presents a number of unclear problems [6]. In-depth insights into how psychophilic yeasts adapt to low temperatures will greatly benefit the understanding of the adaptive mechanism of psychophilic organisms to low-temperature environments.

Numerous psychrophilic yeasts have been discovered in low-temperature regions; they include *Leucosporidium scottii*, *Mrakia psychrophila*, *Mrakia frigida*, *Mrakia blollopis*, *Metschnikowia australis*, *Rhodotorula frigidialcoholis*, *Glaciozyma antarctica*, *Candida psychrophila*, *Meyerozyma caribbica*, etc. [1,7,8,9]. The most widespread species are *Cryptococcus* spp. (Basidiomycetes) [7]. They have the advantages of rapid growth and reproduction, and a simple genetic operation system. Thus, they can be easily monitored at the molecular level. Considering that environments are selective for psychrophilic yeasts, as the environment changes, the diversity of psychrophilic yeasts in the cryosphere shows significantly distinct spatial distribution patterns [1]. However, several psychrophilic yeasts are difficult to culture in the laboratory, and numerous studies are incomplete in their sampling. Therefore, a comprehensive comparison and analysis of psychrophilic yeasts from different sources must be conducted.

With the development of next-generation sequencing technology, whole-genome sequencing technology has become a conventional method used to study psychrophilic microorganisms. Over 50 psychrophilic microorganisms have been sequenced, and most of them are accessible to the public [7]. A substantial amount of genome sequence data have also been obtained from Antarctic metagenomic nucleic acid sequencing [8], deep-sea random whole-genome shotgun sequencing [9], and glacial ice coke sequencing [10]. A functional screening of the Antarctic metagenomic library identified several cold adaptation enzymes, including lipase, esterase, and cellulase [11,12,13]. Moreover, the availability of the *G*. *antarctica* genome has provided significant insights into the cold adaptation strategies acquired by psychrophilic eukaryotes [14]. Several genes of *G. antarctica* are involved in cold adaptation [15]. *Rhodotorula* JG1b was one of the six microorganisms isolated and sequenced from this ice-cemented permafrost [16]. However, current studies on the mechanism of psychrophilic yeasts via whole-genome sequencing are limited to the analysis of single strains. A comparison of multiple psychrophilic yeasts is lacking, and their cold adaptability at the whole-genome level cannot be accurately revealed. In this article, whole genomes of nine reported psychrophilic yeasts (*M. australis* UFMG-CM-Y6158, *M*. *blollopis* SK-4, *M*. *psychrophila* NN053900, *M. frigida* JCM7857, *G. antarctica* PI12, *C. psychrophila*, *L. scottii* PYCC4405, *M. caribbica* MG20W, and the novel extremophilic yeast *R. frigidialcoholis*) [15,17] and three representative mesophilic yeasts (*Saccharomyces cerevisiae* S288C, *Cryptococcus neoformans* var. *neoformans* JEC21, and *Schizosaccharomyces pombe* 972h-) were compared and analyzed. We analyzed the differences in their signal pathways, metabolic regulation, evolution, and psychrophilic genes to better utilize psychrophilic microorganisms for low-temperature applications and studies.

## 2. Material and Methods

### 2.1. Data Collection and Genome Annotation

To extract the genomic information from the nine strains of psychrophilic yeast and the three strains of mesophilic yeast, we manually downloaded the genomes of the nine psychrophilic yeasts and the three mesophilic yeasts from the National Center for Biotechnology Information (NCBI) database (7 August 2022). TBtools v1.100 software was used (https://github.com/CJ-Chen/TBtools (accessed on 10 August 2022) to extract the genomic information. Augustus v3.3 software was used to annotate the genomes [18]. For predicting the secondary metabolites (biosynthetic gene clusters, BGCs), whole-genome sequences were submitted to the antiSMASH database.

### 2.2. Analyses of Metabolic Pathway Enrichment

KofamKOALA (https://www.genome.jp/tools/kofamkoala/ (accessed on 10 August 2022) [19] and KAAS (https://www.genome.jp/tools/kaas/ (accessed on 10 August 2022) [20] were used to analyze the metabolic pathway enrichment, and genes with significant enrichment were selected for further investigation.

### 2.3. Homologous Gene Identification and Domain Analysis

BioEdit v7.0.9.0 and UltraEdit v25.00.0.58 were used to search for psychophilia-related genes in different yeast genomes (e-value: 1 × 10^−5^, hits with best score), and NCBI-BLAST was used to compare and analyze the protein domains.

The constraint-based multiple alignment tool and DNAMAN v9.0.1.116 software were used for multiple sequence alignment and annotation of different psychrophilic-related proteins, and ESPript 3.0 (https://espript.ibcp.fr/ESPript/cgi-bin/ESPript.cgi (accessed on 10 August 2022) was used for mapping the results.

### 2.4. Phylogenomic Analysis

To classify the available AFPs of different psychrophilic yeasts, we downloaded the latest AFPs from NCBI (25 November 2022) and constructed a phylogenomic tree using the neighbor-joining method (bootstrap: 1000). In addition, phylogenomic analyses of whole-genome sequences of different psychrophilic yeasts were inferred with REALPHY 1.12 (https://realphy.unibas.ch/realphy/ (accessed on 15 December 2022). REALPHY utilizes reference whole-genome sequence data to conduct alignments with the query sequences; it is an ultrafast and memory-efficient tool from which phylogenomic trees can be further constructed using PhyML. A maximum likelihood tree was generated using the generalized time-reversible nucleotide substitution model. The resulting tree was visualized and annotated using FigTree v1.4.3.

### 2.5. Modeling of AFPs and Potential Ice-Binding Site Predictions

Through the use of AlphaFold 2.0 (https://www.alphafold.ebi.ac.uk/ (accessed on 21 August 2022), the homology of nine known AFPs in *G. antarctica* PI12 was modeled, and the structural characteristics of these AFPs were analyzed after ensuring the rationality of the three-dimensional structures [21]. With the AFPredictor tool, we further predicted the potential ice-binding sites of different AFPs [22].

## 3. Results

### 3.1. Genome-Wide Characterization of Psychrophilic Yeasts

The genome size of Basidiomycetes (19.39–30.8 Mb) was larger than that of Ascomycetes (10.61–14.35 Mb), and the guanine (G) and cytosine (C) (GC) content of psychrophilic yeasts was generally higher than that of mesophilic yeasts (Table 1). G and C are linked by three hydrogen bonds in DNA, whereas adenine and thymine are linked by two hydrogen bonds. The connections with more hydrogen bonds are less prone to being broken and denatured; thus, DNA molecules with a higher GC content are more stable and less susceptible to denaturation [23]. As a result, psychrophilic yeasts can adapt to low temperatures.

In general, psychrophilic yeasts of the class Ascomycetes contained cryogenic enzymes, such as lipase and glucose amylase, whereas α-amylase was absent from their genomes. α-Amylase only existed in the genomes of psychrophilic yeasts of the class Basidiomycetes. The hypothermic mechanism of the two groups of yeasts may differ in certain aspects. The genomes of psychrophilic yeasts of the class Basidiomycetes contained more secondary metabolite synthesis gene clusters than the class Ascomycetes. Meanwhile, the genome size of psychrophilic yeasts of the class Basidiomycetes was greater than that of the class Ascomycetes. The psychrophilic yeasts of class Basidiomycetes also encoded more catalytic enzymes and may therefore be more environmentally tolerant.

Kyoto Encyclopedia of Genes and Genomes gene enrichment analysis indicated that in the genome of psychophilic yeasts of class Ascomycetes, the number of genes involved in metabolism of peroxisomes, fatty acid metabolism, glycerol ester metabolism, biosynthesis of unsaturated fatty acids, pyruvate metabolism, and glutathione metabolism was significantly greater than that of mesophilic yeasts (Supplementary Figure S1). Therefore, psychophilic yeasts, which can produce glycerol, synthesize unsaturated fatty acids, and remove free oxygen radicals, may be significantly superior to mesophilic yeasts. The number of genes involved in the pentosephosphate pathway and the metabolic pathway involving terpenoid skeleton biosynthesis was comparable with that of Ascomycetes psychrophilic yeasts, whereas the biosynthesis, DNA repair, and glycan biosynthesis pathway of glycosylphosphatidylinositol-anchored substances was significantly superior to that of mesophilic yeasts (Supplementary Figure S1). Glycans are carbohydrate-based polymers associated with cellular protection and storage, in addition to being an integral component of glycoproteins, including cell-surface membrane proteins, such as receptors and adhesion proteins [24]. *R. frigidialcoholis* increases EPS synthesis at 0 °C by overexpressing mannan proteins as an adaptation to the desiccation and freeze–thaw cycles of Antarctica’s University Valley permafrost [17].

### 3.2. Unique Polyunsaturated Fatty Acid (PUFA) Pathways That Benefit Low-Temperature Adaptation

**Figure 1:**
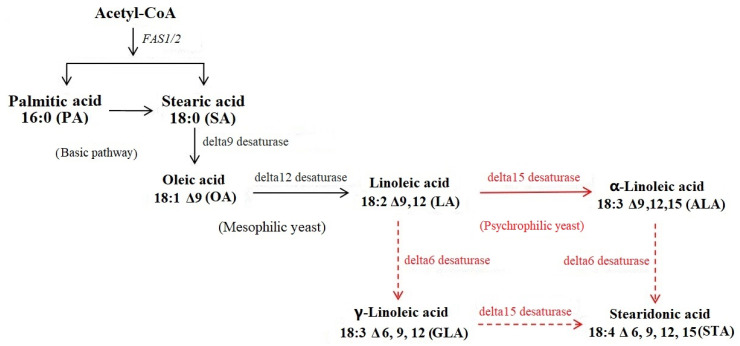
Proposed PUFA pathways in mesophilic and psychrophilic yeasts.

The fluidity of the cell membrane depends on the content of membrane lipids. PUFAs refer to unsaturated fatty acids with two or more carbon double bonds and 18 or more C atoms. Under low-temperature conditions, most microorganisms maintain the fluidity of the cell membrane by changing the content of PUFAs in the cell membrane to ensure the transport of nutrients and various reactions involving membrane proteins [25]. In addition, different organisms exhibit differential preferences for one or more of these pathways because of differences in the selectivity toward the substrate of fatty acid desaturase (FAD), prolonged enzyme lines and enzymes in the cell, and the accumulation of one or more PUFAs accordingly. According to the results (Table 2), different types of FAD-encoding genes (delta 6, delta 9, delta 12, and delta 15) could be found in the genomes of various psychrophilic yeasts, whereas in mesophilic yeasts, the number of FAD-coding genes was significantly less than that of psychrophilic yeasts. Delta 9 (AAW45979.1) and delta 12 (ALO60520.1/AAW42919.1) desaturases was present in the mesophilic yeast strain *C. neoformans*; delta 9 desaturase has been found in the *S. cerevisiae* and *S. pombe* genomes of mesophilic yeasts, whereas the other FAD-coding genes have been lost. Different species or numbers of FADs may be advantageous for psychrophilic yeasts in order to synthesize various types of 18-carbon unsaturated fatty acids (C18:1, C18:2, and C18:3) to adapt to low temperatures versus mesophilic yeasts, which can only synthesize C18:1 or C18:2 unsaturated fatty acids (Table 2). Thus, membrane fluidity is negatively impacted in a low-temperature environment, preventing growth. According to previous studies, oleic acid (delta 9-C18:1) is a ubiquitous unsaturated fatty acid in mesophilic yeasts and is predominantly catalyzed by delta 9 desaturase, whereas linoleic acid (delta (9,12)-C18:2) is secondary in wild-strain *S. cerevisiae* S288C [26,27]. By contrast, given that an 18-carbon fatty acid needs to be introduced by delta 6, 9, 12, and 15 desaturases, forming unsaturated fatty acids with C18 chain lengths, cold-adapted yeast has developed an adaptive mechanism. For instance, the content of α-linolenic acid (C18:3, delta 9,12,15) in the cells of obligate psychrophilic yeasts (*Leucosporidium* spp. and *Mrakia* spp.) was significantly greater than in the cells of obligate psychrophilic yeasts (*p* < 0.05) [28]. In addition, to adapt to low temperatures, the strain *G. antarctica* PI12 significantly upregulated the expression of different FAD genes at different growth temperatures (15 °C, 0 °C, and −12 °C) [15]. At low temperatures, delta 9 (KZ627151.1) and 12 desaturase (AEG19535.1) genes were expressed earlier (6 h) in a culture than delta 6 (GAN_06_180, GAN_11_019) and 15 desaturase (GAN_06_218)-encoding genes. In the later stage (after 48 h), their expression levels were elevated. Consequently, in the early culture stages in a cryogenic environment, *G. antarctica* PI12 can catalyze the introduction of one or two double bonds into the fatty acid chain, such as the synthesis of linoleic acid (C18:2, delta 9, 12), to increase its membrane fluidity for the synthesis of α-linoleic acid (C18:3, delta 9, 12, and 15) and γ-linoleic acid (C18:3, delta 6, 9, and 12) [15]. On the basis of these findings, we hypothesized that the different PUFA synthesis pathways (Figure 1) present in mesothermic yeast and psychrophilic yeast would contain a greater variety of unsaturated fatty acid synthase-coding genes than those found in mesophilic yeasts, especially those capable of catalyzing the synthesis of C18:3 unsaturated fatty acids, and they may express different FAD-encoding genes at various times during the culture process.

### 3.3. Various AFPs Contribute to Low-Temperature Protection

**Figure 2:**
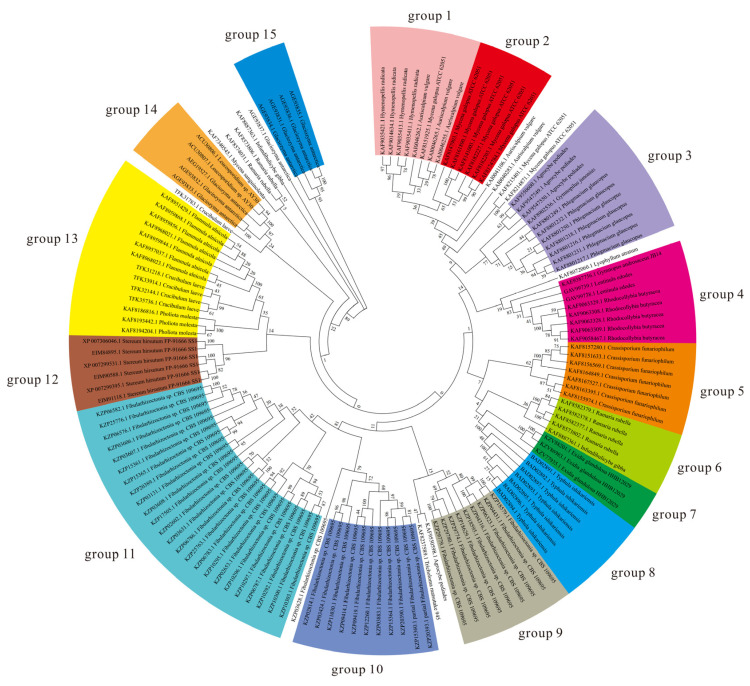
Phylogenomic trees of AFPs.

AFPs/ice-structuring proteins can inhibit the growth of ice crystals and improve the freezing resistance of organisms. They have evolved independently in a wide range of organisms, including bacteria, plants, and fish [29]. Despite their independent origins and diverse folding structures, AFPs bind to a common substrate (ice), albeit on different surfaces and in various orientations. Different anti-cryogenic proteins (AFPs) have different structures, but their surfaces have multiple ice-binding sites, which can be adsorbed onto the ice crystals’ surfaces to inhibit recrystallization and lower the freezing point of the solution, thus helping cells survive at low temperatures [29,30]. An analysis of endemic genes in psychrophilic yeasts revealed that the Antarctic psychrophilic yeast strains *G. antarctica* PI12 and *Leucosporidium* spp. have unique AFPs. The phylogenomic analysis of several AFPs in the strain *G. antarctica* PI12 and AFP genes from other psychrophilic microorganisms revealed that all AFPs can be classified into 15 types, and they showed obvious species-specificity within different psychrophilic yeasts (Figure 2). The evolutionary status of AFPs in the strain *G. antarctica* PI12 is relatively conservative, and they have affinities with psychrophilic yeasts such as the *Leucosporidium* genus (Figure 2). For example, in Group 14 of the AFPs, *G. antarctica* PI12 and *Leucosporidium* sp. AY30 clustered in the same clade (Figure 2). However, the widespread loss of AFPs in the mesophilic yeasts indicates that AFPs are one of the critical psychrophilic genes of the psychrophilic yeasts.

**Figure 3:**
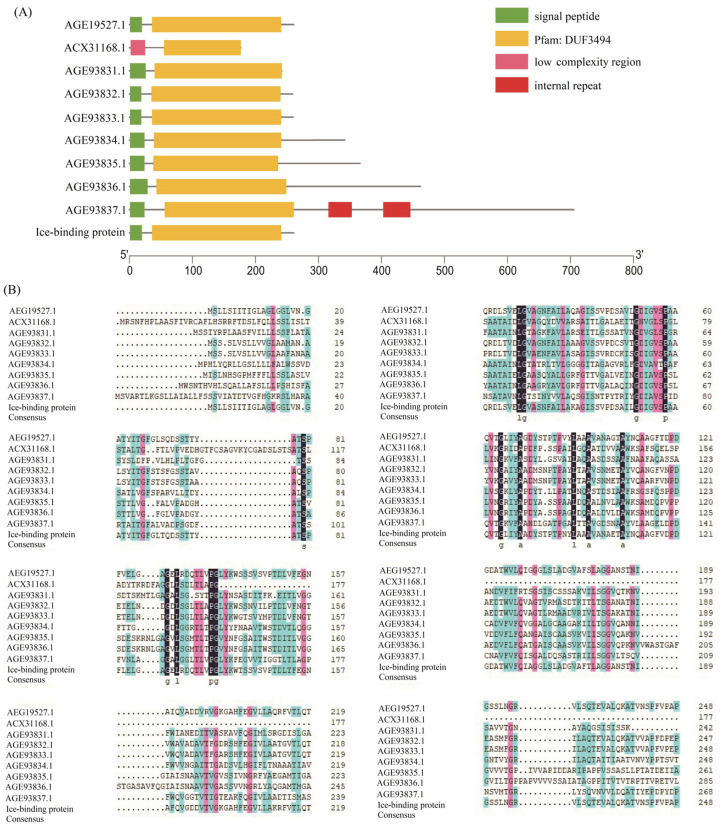
Domains and sequence alignments of AFPs from *G. antarctica* PI12 and *Leucosporidium* spp. (**A**) Domains of AFPs. (**B**) Sequence alignments of AFPs.

In the most researched *G. antarctica* PI12 strains, nine antifreezing genes were present, and they varied, depending on the timing of the low-temperature culture and the response to different temperatures [15]. The level of antifreezing genes in this strain was very low (20.36%), and these genes have not been discovered in any other psychrophilic yeasts. Given that the protein is flexible and catalytically active at low temperatures, the structures such as the active site of the enzyme molecule and its conformation could change at low temperatures and improve the enzyme’s plasticity [31]. One of its main flexible modalities is to reduce the number of arginine (Arg) and proline (Pro) amino acid residues, increase the number of glycine (Gly) amino acids, increase the residual charge on the surface, increase the interactions between protein molecules and solvents, weaken the interactions between anions and cations, and weaken the force of interaction between subunits and domains [32,33]. Various AFPs are members of the DUF3494 gene family (Figure 3A). Numerous proteins containing the domain of uncertain function (DUF) 3494 bind ice crystals and are therefore categorized as ice-binding proteins (IBPs). DUF3494 IBPs are now the most prevalent of all known IBP families [34]. Conservative amino acids in different AFPs include Arg, Pro, and Gly, but when the ratio of Arg and Pro (2%) is lower than the ratio of Gly to Pro (5%), then the proportion of Gly increases significantly (Figure 3B). In proteins, a high ratio of Gly to Pro indicates flexibility and high strength. Therefore, from the perspective of amino acid perspective, the high ratio of Gly/Pro is one of the reasons for the cold-adaptability of AFPs. The conserved Gly clusters (10) are near the functional domain of the structure and contribute to the increased flexibility of the enzyme’s molecule. In addition, its high catalytic efficiency at low temperatures is attributed to the loose and flexible protein structure, which contributes to the high catalytic efficiency of the enzyme [15,35]. In addition, the eight known IBPs in the InterPro database (IPR021884) belong to the DUF3494 gene family and can be roughly divided into three categories (Supplementary Figure S2A,B), which include homologous genes to the AFPs in *G. antarctica* and *Leucosporidium* spp. Overall, these AFPs are less conserved, and the majority of them have conserved G catalytic sites (Supplementary Figure S2C), similar to the G catalytic sites in the nine AFPs from *G. antarctica* (Figure 3B), and may play an important catalytic role in various AFPs. However, over 2000 IBPs in the InterPro database have not been published. Thus, a huge number of unknown AFPs in other psychrophilic yeasts need to be excavated.

**Figure 4:**
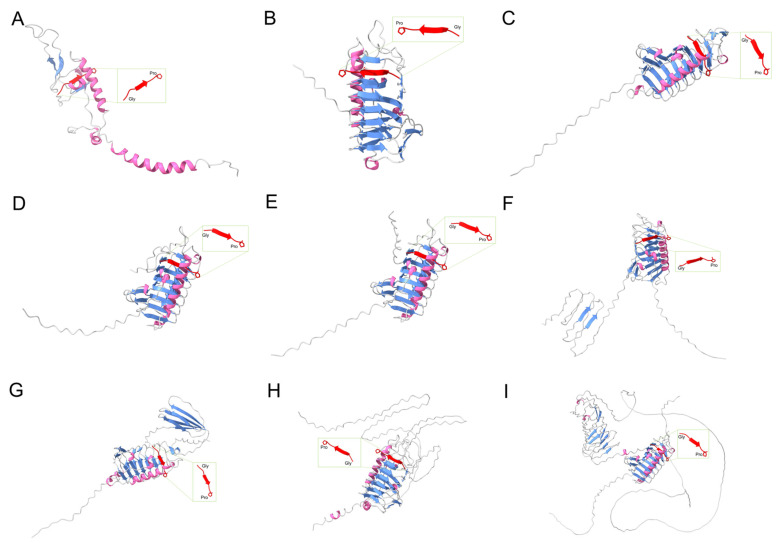
Modeled structure of the AFPs in *G. antarctica* strain PI12. Accession numbers: (**A**) ACX31168.1, (**B**) AEG19527.1, (**C**) AEG93831.1, (**D**) AEG93832.1, (**E**) AEG93833.1, (**F**) AEG93834.1, (**G**) AEG93835.1, (**H**) AEG93836.1, and (**I**) AEG93837.1.

**Figure 5:**
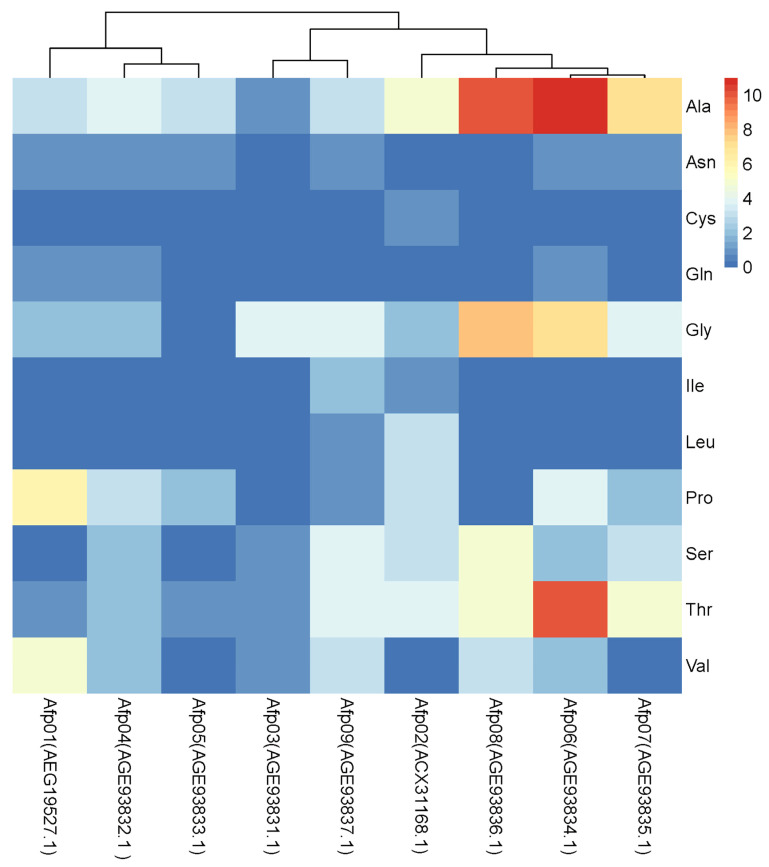
Potential ice-binding sites of different AFPs.

Although anti-cryoprotein genes are primarily found in polar regions, their structure and activity are diverse. In spite of the differences in the primary amino acid sequences between different AFP genes, a number of similarities exist between the tertiary and quadrilateral structures they fold into. As a result, tertiary and quadrilateral structures can bind to different surfaces of ice crystals, thereby inhibiting the ice crystals’ growth or binding to ice-nucleating agents and recrystallizing, and providing low-temperature cell protection [36]. Studies have demonstrated that AFP amino acids possess important physicochemical properties, mainly between hydrogen bonds or water molecules of the hydrophilic side chains of threonine (Thr), glutamine (Gln), and glutamic acid, which bind to the surface of ice. The hydrophilic residues of serine (Ser) may also contribute to their poor freeze-resistant properties [37]. The results of three-dimensional structural analysis of these proteins indicated that the domains of different AFPs are predominantly TIM barrel structures formed by alternating connections of α-helix and β-folded sheets (Figure 4), with conserved Gly and Pro residues in the β-folded sheets, which may be closely related to their properties of low-temperature resistance. The majority of the nine AFPs belong to Class 3 (Figure 4C–I), and their three-dimensional structure is comparable with that of Type IV antifreeze protein, which is a helical beam protein in fish (helix–bundle protein). The structure of AFP01 (ACX31168.1) (Figure 4A) is comparable with that of Type I antifrelaim protein in fish, which consists of alanine-rich and amphipathic molecular helices [38]. Figure 4B shows that the structure of another antifrezenin (AEG19527.1) is distinct from that of fish and insects and may be unique to psychrophilic yeasts. Furthermore, ice-binding sites, such as alanine (Ala), Gly, and Pro, and hydrophilic residues, such as Thr, are prevalent in the nine anti-cryoproteins, which suggests that the AFPs in psychrophilic yeasts, including *G. antarctica* and *Leucosporidium* spp., may exert their antifreeze effect mainly through hydrogen bonds or hydrogen bonds between water molecules and the hydrophilic side chains of Thr, or by binding to the ice’s surface. In addition, the results in Figure 5 show that the hydrophilic residues Thr, Gly, and Ala are mainly enriched in AFP06 and AFP07, with fewer hydrophilic residues of Ser, which can relieve the osmotic stress caused by freezing and dehydration, and maintain the integrity of the cell membrane’s structure. Meanwhile, AFP01 (AEG19527.1) has two hydrophilic residues of Thr and Gln but no hydrophilic residue of Ser (Figure 5), which indicates a significant advantage for antifreezing. Therefore, the different numbers and types of ice-binding sites in various AFPs may assist in the survival of psychrophilic yeasts under low temperatures (Figure 5) [36,39].

## 4. Conclusions

This study demonstrated that psychrophilic yeasts differ from mesophilic yeasts in terms of their psychrophilic mechanisms at the genomic level and have significant differences in their metabolic pathways, genome composition, and psychrophilic genes. By changing the content of PUFAs in the cell membrane, psychophilic yeasts can maintain their cell membranes’ fluidity and alter the molecular conformation and amino acid composition of the enzymes’ proteins, resulting in increased protein flexibility. This characteristic possibly ensures their low-temperature adaptations. Our results showed that psychrophilic yeasts are closely associated with their unique genomes.

## Figures and Tables

**Table 1:** Genomic information of different yeasts.

Strains	Isolations	Accession No.	Sequence Length (Mb)	GC Content (%)	BGCs			CAEs		
					Terpene	Nrps	Nrps-like	Lipase	Glucoamylase	α-Amylase
*L. scottii*	Southern Ocean (sea water)	GCA_003054985.1	26.75	0.59	1	2	2	1	0	1
*M. psychrophila*	China: Hailuogou, Sichuan (alpine glacier soil)	GCA_001889225.1	27.77	0.54	1	0	1	0	0	1
*M. frigida*	Not applicable	GCA_001600395.1	28.62	0.54	1	0	2	0	0	1
*M. blollopis*	Antarctica: East Antarctica, Skarvsnes ice-free area	GCA_000950635.1	30.48	0.54	1	0	2	0	0	1
*G. antarctica*	Sea ice near Casey Research Station, Antarctica	GCA_002917775.1	20.03	0.60	2	1	1	0	0	1
*R. frigidialcoholis*	Missing	GCA_001541205.1	19.39	0.61	2	1	1	1	0	1
C. neoformans *	Not applicable	GCF_000091045.1	19.05	0.49	2	0	2	1	1	1
*M. australis*	Antarctica: Admiralty Bay, King George island	GCA_002073855.1	14.35	0.47	1	0	1	1	1	0
*C. psychrophila*	Antarctica	GCA_900186205.1	11.24	0.37	0	0	1	1	1	0
*M. caribbica*	Reclamation soil	GCA_000755205.1	10.61	0.44	1	0	1	1	1	0
S. cerevisiae *	Not applicable	GCF_000146045.2	12.16	0.38	1	0	0	1	1	0
S. pombe *	Not applicable	GCF_000002945.1	12.60	0.36	0	0	1	1	1	1

* Mesophilic yeasts. BGCs, biosynthesis gene clusters; CAEs, cold-active enzymes.

**Table 2:** FADs of different yeasts.

Strains	Number of PUFAs			
	Delta 6 Desaturase	Delta 9 Desaturase	Delta 12 Desaturase	Delta 15 Desaturase
*L. scottii*	1	1	1	0
*M. psychrophila*	0	1	1	1
*M. frigida*	0	1	1	1
*M. blollopis*	0	1	1	1
*G. antarctica*	1	1	1	1
*R. frigidialcoholis*	1	1	1	1
C. neoformans *	0	1	1	0
*M. australis*	1	1	1	1
*C. psychrophila*	1	1	1	1
*M. caribbica*	1	1	1	1
S. cerevisiae *	0	1	0	0
S. pombe *	0	1	0	0

* Mesophilic yeasts.

## Data Availability

The data of this systematic review are available in the manuscript.

## Supplementary Materials

The following supporting information can be downloaded at https://www.mdpi.com/article/10.3390/genes14010158/s1. Figure S1: Gene enrichment of different fungi: Ascomycetes (A) and Basidiomycetes (B); Figure S2: The phylogenetic tree (A), domains (B), and sequence alignments (C) of the reported antifreeze proteins (AFPs).
