# Supplementary material for: Psychrophilic Yeasts: Insights into Their Adaptability to Extremely Cold Environments
Source: Genes (Basel). 2023 Jan 6;14(1):158. doi: 10.3390/genes14010158 (PMC9859383; doi:10.3390/genes14010158)
Supplement: Supplementary file 1 [file genes-14-00158-s001.zip › genes-2056839-supplementary.pdf]

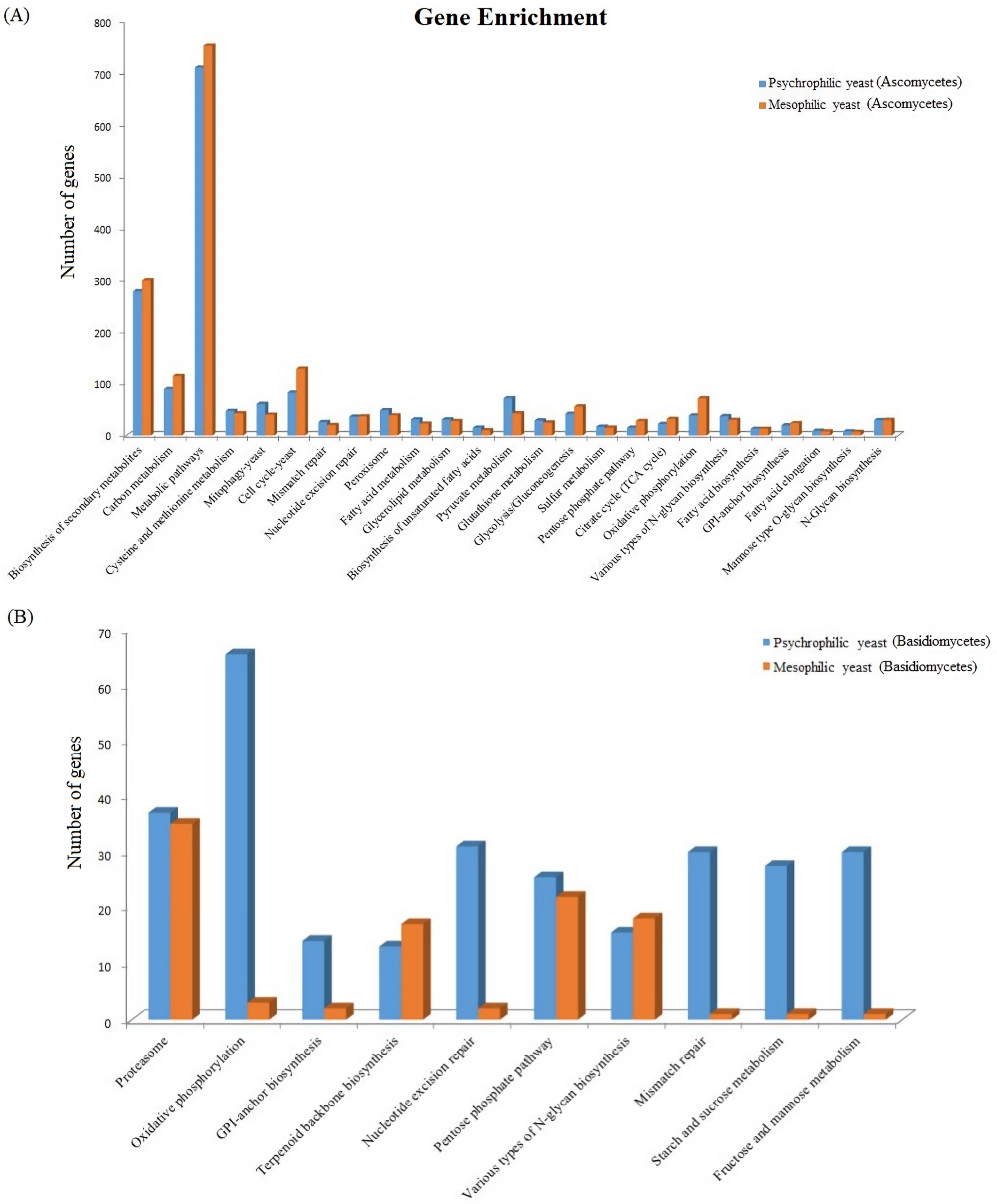

Supplementary Figure S1. Gene enrichment of different fungi: Ascomycetes (A) and Basidiomycetes (B).

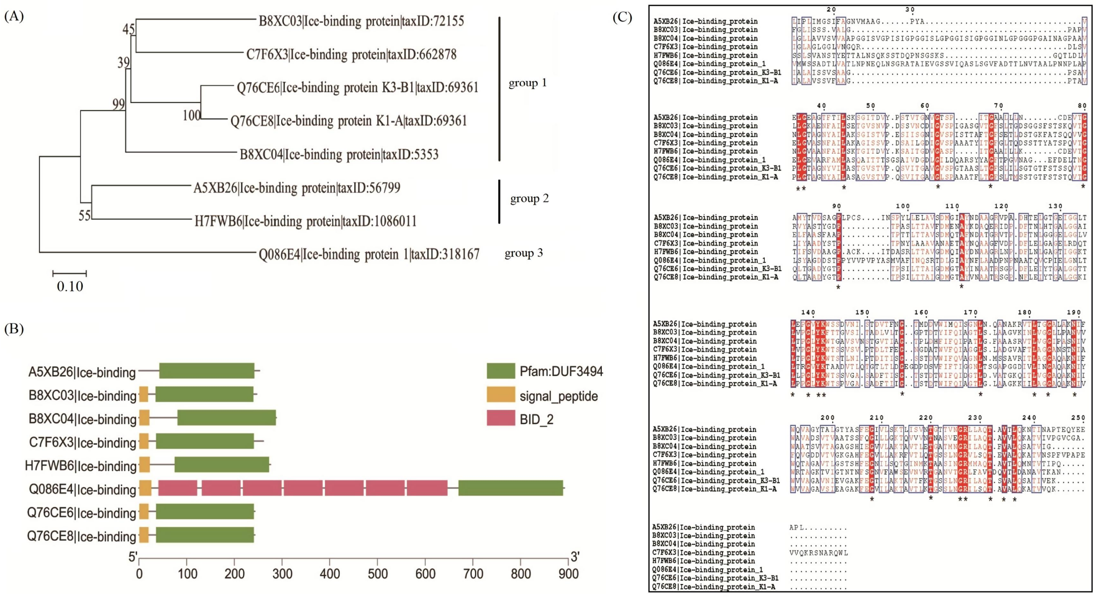

Supplementary Figure S2. The phylogenetic tree (A), domains (B), and sequence alignments (C) of the reported antifreeze proteins (AFPs). Highly conserved residues are highlighted in red
